# Similarities and Differences between the Pathogenesis and Pathophysiology of Diastolic and Systolic Heart Failure

**DOI:** 10.1155/2013/824135

**Published:** 2013-12-29

**Authors:** Kazuo Komamura

**Affiliations:** Cardiovascular Division, Hyogo College of Medicine, 1-1 Mukogawa-cho, Nishinomiya, Hyogo 663-8501, Japan

## Abstract

Pathophysiology of heart failure has been considered to be a damaged state of systolic function of the heart followed by a state of low cardiac output that is, systolic heart failure. Even if systolic function is preserved, left ventricular filling in diastole can be impeded and resulted in elevation of filling pressure and symptoms of heart failure. This kind of heart failure is called diastolic heart failure. Nowadays, diastolic heart failure is referred to as heart failure with preserved ejection fraction (HFpEF), whereas systolic heart failure is referred to as heart failure with reduced ejection fraction (HFrEF). In this paper, the similarities and differences between the pathogenesis and pathophysiology of diastolic and systolic heart failure were reviewed. Although diastolic heart failure is a common condition of heart failure worldwide, its pathophysiology has not been sufficiently elucidated. This is thought to be the most significant reason for a lack of established treatment methods for diastolic heart failure. We hope to proceed with future studies on this topic.

## 1. Introduction

Pathophysiology of heart failure has been considered to be a damaged state of systolic function of the heart followed by a state of low cardiac output (systolic heart failure). However, even if systolic function is preserved, left ventricular filling in diastole is impeded due to various factors. This condition leads to congestive heart failure due to the rise in left ventricular end-diastolic pressure and the decrease in cardiac output. This kind of pathophysiology is now known as diastolic heart failure [1, 2]. In recent years, diastolic heart failure caused by the affected left ventricle has become a clinical issue [3]. Nowadays, diastolic heart failure is referred to as heart failure with preserved ejection fraction (HFpEF), whereas systolic heart failure is referred to as heart failure with reduced ejection fraction (HFrEF). This is because evaluating accurate pathophysiology and diagnosis of diastolic heart failure is in fact difficult.

## 2. Diastolic Dysfunction

Diastole of the left ventricle is composed of isovolumic relaxation and ventricular filling. Relaxation of the left ventricle is an active process that occurs as a result of energy-dependent uptake of intracellular calcium by the sarcoplasmic reticulum, whose concentration has risen during the systolic phase.

Relaxation of the left ventricle is impaired in a disease state caused by energy metabolism disorders or calcium-handling abnormalities such as myocardial ischemia and myocardial hypertrophy. Left ventricular filling phase abnormality, namely, elevation of left ventricular stiffness, influences left ventricular flow dynamics during filling phase and is commonly caused by myocardial fibrosis or hypertrophy.

When left ventricular diastolic function is impaired, cardiac output is reduced because the left ventricle is not filled enough in diastole due to left ventricular inflow obstruction. By contrast, to compensate for reduced cardiac output, increasing the inflow pressure to the left ventricle (and consequently left ventricular end-diastolic pressure) becomes necessary, which in turn increases left atrial pressure. As a result, left ventricular dysfunction easily and directly causes pulmonary congestion.

**Figure 1:**
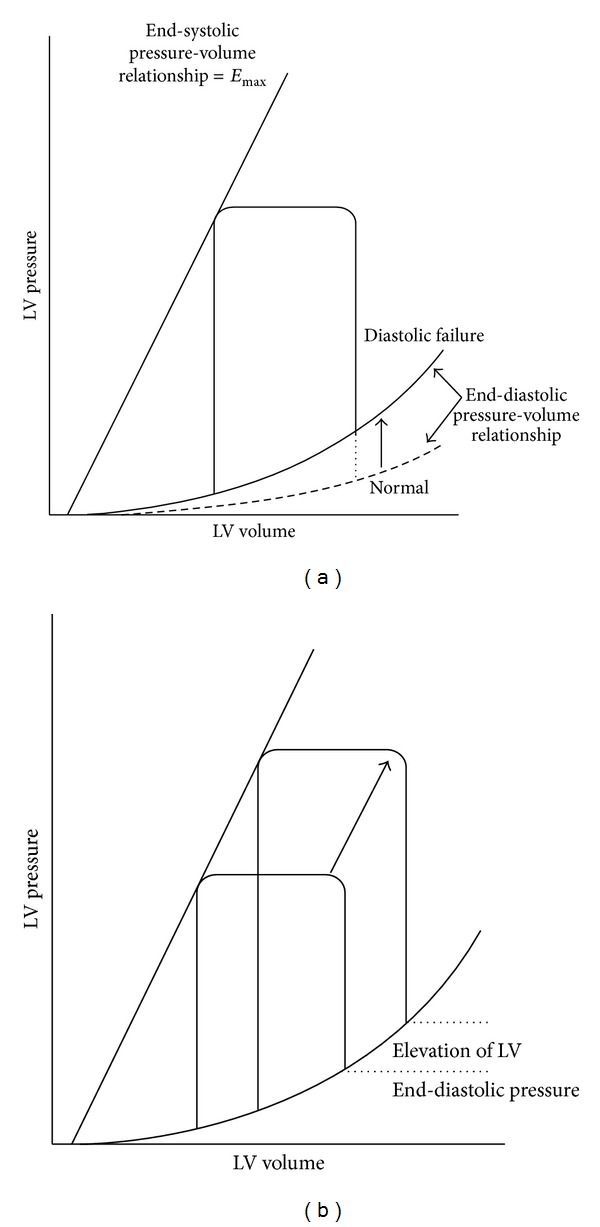
(a) The end-systolic pressure-volume relationship is the same as a normal heart in diastolic heart failure, but the end-diastolic pressure-volume relationship shifts upwards. As a result, left ventricular end-diastolic pressure rises. (b) In pathologies with diastolic dysfunction, when an abrupt increase in blood pressure occurs, the pressure-volume loop shifts to the upper right without decrease in *E*_max⁡_. Therefore, pulmonary congestion is induced as a result of the significant increase in left ventricular end-diastolic pressure. LV: left ventricular.

The end-systolic pressure-volume relationship is the same as a normal heart in diastolic heart failure, but the end-diastolic pressure-volume relationship shifts upwards (Figure 1(a)) [3]. As a result, left ventricular end-diastolic pressure rises. In pathologies with diastolic dysfunction, when an abrupt increase in blood pressure occurs, the pressure-volume loop shifts to the upper right without decrease in *E*_max⁡_ (absolute index of contractibility). Therefore, pulmonary congestion is induced as a result of the significant increase in left ventricular end-diastolic pressure (Figure 1(b)).

**Figure 2:**
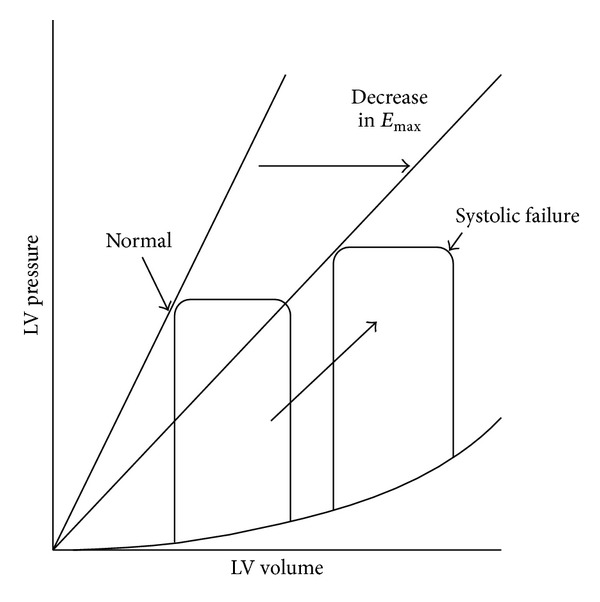
In systolic dysfunction, LV contractile function decreases and *E*_max⁡_ gets smaller. Meanwhile, the end-diastolic pressure-volume relationship shifts downwards rather than remaining unchanged. To maintain cardiac output, the pressure-volume loop shifts right due to increase in preload. Therefore, the LV pressure-volume loop operates on the steep part of the end-diastolic pressure-volume curve, consequently causing end-diastolic pressure to rise. LV: left ventricular.

By contrast, in systolic dysfunction, left ventricular contractile function decreases and *E*_max⁡_ gets smaller (Figure 2). Meanwhile, the end-diastolic pressure-volume relationship shifts downwards rather than remaining unchanged. To maintain cardiac output, the pressure-volume loop shifts right due to increase in preload. Therefore, the left ventricular pressure-volume loop operates on the steep part of the end-diastolic pressure-volume curve, consequently causing end-diastolic pressure to rise.

## 3. Are Diastolic Dysfunction and Systolic Dysfunction Separate Diseases?

**Figure 3:**
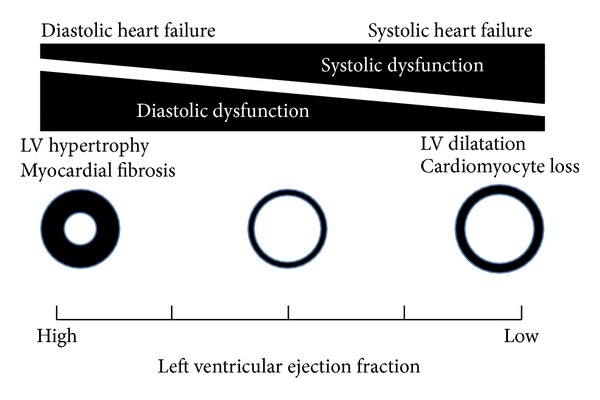
Diastolic and systolic heart failure are not considered to be independent and separate entities. Heart failure is a single continuous disease spectrum and systolic and diastolic heart failure are phenotypes at two extremes.

In diastolic dysfunction, contractility of whole left ventricle is considered normal. However, the contractile velocity in systole measured with tissue Doppler decreased in both systolic and diastolic dysfunction [4]. Furthermore, local contractility in longitudinal direction is known to be impaired locally in diastolic heart failure [5]. Recent findings suggest that contractility decreases even in diastolic heart failure in myocardium level. By contrast, diastolic function is also impaired in systolic heart failure and has been shown to decrease exercise tolerance and be one of the determinants of prognosis [6]. Therefore, diastolic and systolic heart failure are not considered to be independent and separate entities. The single syndrome hypothesis of heart failure is therefore advocated (Figure 3) [1]. In that hypothesis, heart failure is a single continuous disease spectrum and systolic and diastolic heart failure are phenotypes at two extremes.

Thus, there is the “grey zone” in diagnosing HFrEF with LVEF of 45 to 50% or 45 to 55%. In other words, some of HFpEF with LVEF of 45 to 55% might be diagnosed as HFrEF rather than HFpEF. As shown in Figure 3, a phenotype of heart failure comprised of some extent of systolic dysfunction and some extent of diastolic function. Heart failure with LVEF of 45 to 55% would be located in the middle of the continuum of disease spectrum.

By contrast, some researchers have advocated that diastolic function is not something that should only be noted in the pathogenesis of diastolic heart failure, but should be widely viewed as a determinant of pathophysiology in heart failure [7]. Heart dysfunction that occurs as a result of heart disease causes diastolic dysfunction. Among such cases there exist patients with concurrent systolic dysfunction. Furthermore, a portion of patients with heart dysfunction clinically exhibit symptoms of heart failure. Among them, those with significant systolic dysfunction where the main pathology is systolic heart failure, and diastolic dysfunction, are said to have diastolic heart failure.

## 4. The Diagnostic Criteria for Diastolic Heart Failure 

Definition of systolic heart failure is reduction of the left ventricular ejection fraction. Thus, its diagnosis is quite easy. By contrast, diagnosis of diastolic heart failure is difficult since there are no simple and reliable criteria. Therefore, diastolic heart failure can be clinically diagnosed when clinical symptoms and findings of heart failure are exhibited and decrease in left ventricular ejection fraction is none or minimal.

The American College of Cardiology Foundation and the American Heart Association define diastolic heart failure as a condition having the typical signs and symptoms of heart failure with a normal left ventricular ejection fraction, without valvular abnormalities on echocardiography [8]. Vasan and Levy define diastolic heart failure as (1) exhibiting clinical symptoms of congestive heart failure, (2) having normal left ventricular systolic function during congestive heart failure (left ventricular ejection fraction of 45 to 50% and above), and (3) having left ventricular diastolic dysfunction. Those that meet all these items and who have congestive heart failure that is not caused by valvular heart disease, cor pulmonale, or primary volume overload are considered as “definite diastolic heart failure (definite DHF)” cases. Currently, accurate diagnosis of (3) needs cardiac catheterization, which could be skipped in a common clinical situation.

Diastolic heart failure is strongly suspected (probable DHF) when conditions (1) and (2) are met [9]. When diagnosing diastolic heart failure, it is important to perform a careful exclusion of valvular heart disease, pericardial disease, right heart failure, intracardiac tumor, congenital heart disease, and high-output cardiac failure. Zile et al. demonstrated that diastolic functional abnormalities caused an increase in left ventricular filling pressure and clinical symptoms of congestive heart failure using cardiac catheterization for patients with heart failure with preserved ejection fraction and patients with diastolic heart failure [10, 11]. The currently accepted criteria for diagnosis of diastolic heart failure are essentially a clinical diagnosis. Thus, it is important to understand that there are possibilities that diastolic heart failure means somewhat broader range than what diastolic heart failure exactly stands for.

## 5. Diastolic Heart Failure from the Perspective of Clinical Features

According to reports by Owan et al. and Bhatia et al., roughly half of hospitalizations for heart failure are due to diastolic heart failure [12, 13]. Compared with systolic heart failure, diastolic heart failure is seen more often in the elderly and women and accompanied by hypertension and anemia. Comorbidity rate of obesity, diabetes, and chronic kidney disease (CKD) in diastolic heart failure is high, but not particularly higher than in systolic heart disease. In general, both diastolic and systolic heart failure exhibit distinctive subjective symptoms and objective findings of heart failure including dyspnea, edema, and malaise. Symptoms of diastolic heart failure typically include dyspnea due to pulmonary congestion, particularly shortness of breath, paroxysmal atrial fibrillation, and rapidly developing dyspnea induced by tachycardia, all of which are common initial symptoms. By contrast, in systolic heart failure, symptoms and signs due to general malaise and organ hypoperfusion associated with decreased cardiac output are frequently seen.

The main differences between diastolic and systolic heart failure are the presence of contractile dysfunction and left ventricular remodeling (Table 1). In systolic heart failure, progressive ventricular dilatation, or eccentric cardiac hypertrophy, can be seen. By contrast, diastolic heart failure exhibits concentric ventricular remodeling without dilatation or concentric cardiac hypertrophy. The tissue Doppler E/E′ ratio (early mitral inflow peak velocity/early diastolic mitral annular velocity) is an established diastolic function index that is not affected by hemodynamic load, but increases in both diastolic and systolic heart failure. Pattern of pulmonary vein flow and E/A (ratio of early to late mitral inflow peak velocity) are also established as major diastolic functional indices, though they are rather dependent on hemodynamic status [14].

In recent years, several reports on important association of various biomarkers for heart failure and subtypes of heart failure have been published [15–19]. According to those reports, plasma B-type natriuretic peptide (BNP) concentration in patients with HFpEF is lower than that in patients with HFrEF [15]. Nonetheless, at a given level of BNP, the prognosis in patients with HFpEF is as poor as in those with HFrEF [15]. Not only BNP and N-terminal proBNP (NT-proBNP) but also high sensitive troponin T (hsTnT) was significantly associated with the risk for HFrEF [16]. On the other hand, growth differentiation factor 15 (GDF15), cystatin C, and urinary albumin excretion were significantly associated with the risk for HFpEF [16, 17]. Researchers suggest that biomarkers relevant to myocardial injury (TnT) and myocardial stress (BNP, NT-proBNP, and midregional proadrenomedullin) have significant relation with HFrEF [17–19] and biomarkers relevant to extracellular matrix remodeling (Galectin-3 and GDF15) have significant relation with HFpEF [17–19].

## 6. The Pathogenesis of Diastolic Heart Failure

The histological features of systolic heart failure include myocardial hypertrophy, loss of myocardial cells, and restructuring of the extracellular matrix. Meanwhile, significant myocardial fibrosis together with myocardial hypertrophy is typical in diastolic heart failure. Myocardial fibrosis is thought to be the main factor in increased stiffness [14].

Mechanical stimulation to the myocardium is the main factor of myocardial hypertrophy, while myocardial fibrosis may be caused by humoral factors such as various cytokines, growth factors, and hormones. In hypertensive HFpEF model rats, oxidative stress was increased and angiotensin II was produced within the arterial walls due to high blood pressure. This resulted in fibroblast activation and increased production of transforming growth factor-*β* via macrophage infiltration and activation mediated by monocyte chemotactic protein-1. The resulting perivascular inflammation is reported to be the cause of reactive fibrosis of myocardium [14–16]. In Dahl salt-sensitive rat HFpEF models, it was found that endothelin, together with angiotensin II, is an important mediator of myocardial fibrosis [17].

In addition to the quantitative increase in collagen and distribution abnormalities, qualitative changes are also involved in increased myocardial stiffness caused by fibrosis. In Dahl salt-sensitive rat HFpEF models, the increase in the ratio of stiff type I collagen to type III collagen, which is highly distensible, and increased collagen cross-linking are reported to important factors of increased myocardial stiffness [18].

In diastolic heart failure, myocardial stiffness of cardiomyocytes per se also increases. Detailed mechanism for this remains unclear but is thought to be due to changes in structural proteins associated with myocardial hypertrophy. Titin, which is a giant sarcomeric protein, acts as a molecular spring and plays a large part in the distensibility of cardiomyocytes during diastole. However, in diastolic heart failure, compared with systolic heart failure, the ratio of large, distensible N2A isoforms small, rigid N2B isoforms to was found to decrease [19].

## 7. Therapeutic Options

To date, angiotensin converting enzyme inhibitors (ACEIs), angiotensin receptor blockers (ARB), beta-blockers, and statins have been tried for HFpEF. Although they are authorized for optimal treatment for HFrEF, none of them can provide the optimal treatment for HFpEF [2]. ACEI perindopril was tested for HFpEF patients for the first time in PEP–CHF trial [20]. It showed no difference in mortality and/or hospitalization rate for heart failure. CHARM-preserved trial, in which ARB candesartan was tested for cardiovascular mortality and heart failure hospitalizations, failed to demonstrate a beneficial effect on cardiovascular death but observed fewer heart failure hospitalizations in the candesartan group [21]. I-PRESERVE was so far the largest trial for HFpEF using ARB irbesartan or placebo. Mortality or hospitalization rate for cardiovascular causes was again not improved by irbesartan [22]. In OPTIMIZE-HF registry, discharge use of beta-blockers exerted no effect on one-year mortality or hospitalization rate in HFpEF patients [23]. A preliminary report suggested statin therapy to be beneficial in HFpEF with lower mortality rate [24].

A neutral outcome in HFpEF compared with a positive outcome in HFrEF, as occurred with ACEIs, ARBs, and beta-blockers, might be compatible with flawed study design. However, a positive outcome in HFpEF compared with a neutral outcome in HFrEF, as occurred with statins, can no longer be attributable to study design but supports different signal transductions driving myocardial remodeling in HFpEF and HFrEF [25].

Several compounds seem to be promising for drug target of HFpEF. Phosphodiesterase 5 inhibitors (PDE5I) increase cGMP level, attenuate adrenergic stimulation, reduce ventricular-arterial stiffening, antagonize maladaptive chamber remodeling, improve endothelial function, and reduce pulmonary vascular resistance [26–30]. The PDE5I sildenafil is currently being tested in the RELAX trial, which evaluates the effects of PDE5I on exercise capacity, functional status, and ventricular function [31]. A preliminary open-label trial documented improvements in exercise capacity and the E/E′ ratio in HFpEF treated with spironolactone [32]. Aldosterone antagonists are currently being actively investigated for HFpEF in the clinical situation. Chamber stiffness is altered by the extracellular matrix like collagen. Alagebrium chloride (ALT-711) is a novel agent that breaks glucose cross-links and improves ventricular and vascular compliance in animal experiments and reduces blood pressure and vascular stiffness in humans [33, 34]. Small open-label trial revealed that ALT-711 was associated with reduced LV mass and improved diastolic filling [35]. Diastolic dysfunction in HFpEF may be related to abnormalities in energy availability or utilization in myocytes [36–38]. Recently, abnormal ATP phosphocreatine shuttle kinetics in HFpEF was demonstrated, and similar results were recently also reported [37, 39]. Currently, a novel therapy targeting energy utilization is under investigation [40].

## 8. Conclusion

Despite the fact that diastolic heart failure is a common condition of heart failure, its precise definition still remains unclear. Therefore, the similarities and differences between the pathogenesis and pathophysiology of diastolic and systolic heart failure have not been sufficiently elucidated. This is thought to be the most significant reason for a lack of established treatment methods for diastolic heart failure. We hope to proceed with future studies on this paper.

## Figures and Tables

**Table 1:** Comparison of systolic and diastolic heart failure.

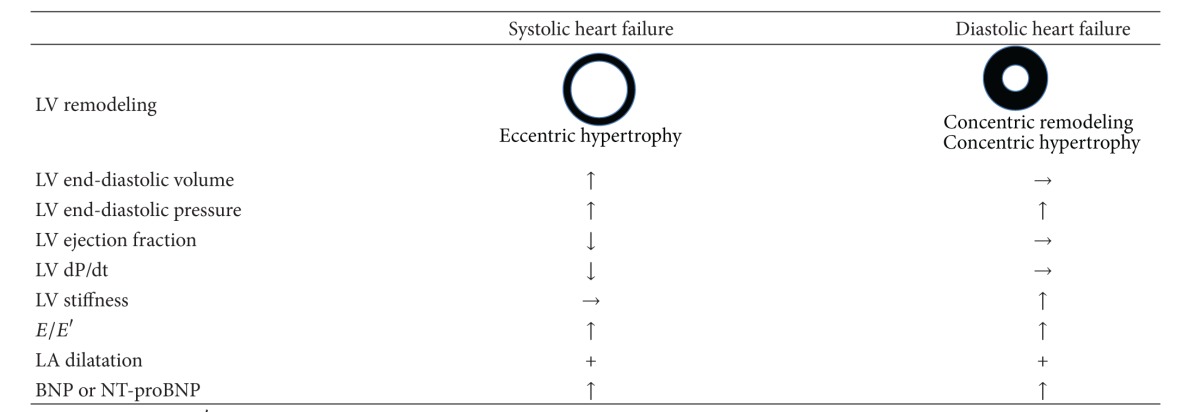

LV: left ventricular; *E*/*E*′: early mitral flow velocity/early diastolic mitral annular velocity ratio; LA: left arterial; BNP: brain natriuretic peptide.
